# Resting heart rate as a marker for identifying the risk of undiagnosed type 2 diabetes mellitus: a cross-sectional survey

**DOI:** 10.1186/1471-2458-14-1052

**Published:** 2014-10-09

**Authors:** Yu-qian Li, Chang-qing Sun, Lin-lin Li, Ling Wang, Yi-rui Guo, Ai-guo You, Yuan-lin Xi, Chong-jian Wang

**Affiliations:** Department of Clinical Pharmacology, School of Pharmaceutical Science, Zhengzhou University, Zhengzhou, Henan PR China; Department of Epidemiology and Biostatistics, College of Public Health, Zhengzhou University, 100 Kexue Avenue, Zhengzhou, 450001 Henan PR China; Department of Disease Control and Prevention, Henan Provincial Center for Disease Control and Prevention, Zhengzhou, Henan PR China

**Keywords:** Marker, Resting heart rate, Type 2 diabetes mellitus

## Abstract

**Background:**

Fast resting heart rate might increase the risk of developing type 2 diabetes mellitus (T2DM). However, it is unclear whether resting heart rate could be used to predict the risk of undiagnosed T2DM. Therefore, the purposes of this study were to examine the association between resting heart rate and undiagnosed T2DM, and evaluate the feasibility of using resting heart rate as a marker for identifying the risk of undiagnosed T2DM.

**Methods:**

A cross-sectional survey was conducted. Resting heart rate and relevant covariates were collected and measured. Fasting blood samples were obtained to measure blood glucose using the modified hexokinase enzymatic method. Predictive performance was analyzed by Receiver Operating Characteristic (ROC) curve.

**Results:**

This study included 16, 636 subjects from rural communities aged 35–78 years. Resting heart rate was significantly associated with undiagnosed T2DM in both genders. For resting heart rate categories of <60, 60–69, 70–79, and ≥80 beats/min, adjusted odds ratios for undiagnosed T2DM were 1.04, 2.32, 3.66 and 1.05, 1.57, 2.98 in male and female subjects, respectively. For male subjects, resting heart rate ≥70 beats/min could predict undiagnosed T2DM with 76.56% sensitivity and 48.64% specificity. For female subjects, the optimum cut-off point was ≥79 beats/min with 49.72% sensitivity and 67.53% specificity. The area under the ROC curve for predicting undiagnosed T2DM was 0.65 (95% *CI*: 0.64-0.66) and 0.61(95% *CI*: 0.60-0.62) in male and female subjects, respectively.

**Conclusions:**

Fast resting heart rate is associated with an increased risk of undiagnosed T2DM in male and female subjects. However, resting heart rate as a marker has limited potential for screening those at high risk of undiagnosed T2DM in adults living in rural areas.

## Background

Type 2 diabetes mellitus (T2DM) is a major public health problem causing significant morbidity and mortality in both developed and developing countries [1, 2]. Previous research indicated that the aetiology of T2DM is complex and there are several risk factors associated with the disease occurrence [3, 4]. In addition, T2DM has a latent nature in a large proportion of cases. Therefore, identifying factors that may predict risk of T2DM, especially undiagnosed T2DM is an important step toward improved understanding and prevention of this major disease in high-risk populations [5, 6]. Blood glucose measurement with continuous monitoring is the standard method used to identify and diagnose T2DM [7], but it is not routinely used in resource limited settings, especially in rural areas of developing countries. Many individuals with T2DM remain undetected due to lack of affordability and availability of diagnostic equipment [8]. Therefore, a simple and inexpensive screening approach is necessary to identify those at high risk of undiagnosed T2DM in resource limited countries and areas.

Pulse rate is an easy measurement but important indicator of cardiovascular diseases. Many studies have shown that fast resting heart rate is an important marker of increased morbidity and mortality among people with cardiovascular diseases [9–12], which indicated that sympathetic over-activity may be a contributing factor to the development of cardiovascular diseases and diabetes [13]. Previous epidemiologic studies have shown that fast resting heart rate is an independent risk factor for the development of T2DM in Japanese individuals [14] and other populations [15–17]. These findings suggest that fast resting heart rate might have an increased risk for the development of T2DM. However, data from rural areas are limited and mainly from urban areas and developed countries. In addition, no study has been published exploring whether resting heart rate could be used as a marker to identify the risk of undiagnosed T2DM. Therefore, the purposes of the study were to examine and confirm the association between resting heart rate and undiagnosed T2DM, and evaluate the feasibility of using resting heart rate as a marker to screen those at high risk of undiagnosed T2DM in adults living in rural areas.

## Methods

### Study population and samples

The study was a population-based, cross-sectional survey, and subjects were selected cluster randomly from eligible candidates listed in the residential registration record from the rural district of Luoyang City in Henan Province of China, which has a population of 99.67 million by 2009, including rural population of 62.09 million, and urban population 37.58 million. The eligibility of the candidate was defined as those who were stable residents for at least 10 years in the areas aged 35–78 years. Exclusion factors included participants with the following conditions within the past six months: 1) individuals with known diabetes, severe psychological disorders, physical disabilities, cancer, chronic kidney disease, Alzheimer’s disease, or dementia; or 2) tuberculosis, acquired immune deficiency syndrome (AIDS), or other infectious diseases. The relevant information of the disease was obtained by the local rural healthcare practitioners. After known diabetes (n = 730), cancer (n = 48), chronic kidney disease (n = 156), physical disabilities (n = 9), Alzheimer’s disease (n = 7), tuberculosis (n = 13), and other infectious diseases (n = 11) were excluded, 16,754 subjects who met the criteria were enrolled in the study. Of the eligible participants, 118 (0.70%) subjects were excluded because of missing information on resting heart rate (n = 71), blood glucose (n = 47). Ultimately, 16,636 subjects were selected for the present analysis. The procedure of the study was approved by the Zhengzhou University Medical Ethics Committee, and written informed consent was obtained from all participants.

### Data collection and laboratory measurement

Data were collected by specially trained physicians and public health workers using standardized methods with stringent levels of quality control. A standardized questionnaire addressed information regarding demographics (age, sex, and residence), socioeconomic status (occupation, educational level, marital status, and individual annual income), family and individual disease history (hypertension, diabetes, heart disease, cancer, chronic kidney disease, stroke, tuberculosis, and AIDS), dietary and lifestyle (smoking, drinking, fat intake, vegetable and fruit intake, and physical activity). Details about the measurement methods and definitions of each variable have been published previously [18, 19]. In brief, dietary intake data were collected from each subject using three-day diet records, and physical activity level was assessed by the International Physical Activity Questionnaire (IPAQ) [20]. Waist circumference (WC) was measured twice at the mid-point between the lowest rib and the iliac crest to the nearest 0.1 cm, after inhalation and exhalation. In addition, the interview included questions related to the diagnosis and treatment of diabetes. Women were asked whether diabetes had been diagnosed during pregnancy and/or when they were not pregnant.

Blood pressure and pulse rates were measured using HEM-770A sphygmomanometer in the sitting position three times according to the American Heart Association’s standardized protocol [21]. Participants were advised to avoid alcohol, cigarette smoking, coffee, tea, and excessive exercise for at least 30 minutes before their blood pressure measurement. Pulse pressure is the difference between systolic and diastolic blood pressure. The normal range of pulse pressure is from 20 to 60 mmHg, and the abnormal ranges is defined as a pulse pressure higher than 60 mmHg or lower than 20 mmHg according to the Chinese Hypertension Prevention Guide. Resting heart rate was classified into four categories: <60, 60–69, 70–79, and ≥80 beats/min.

Blood specimens were collected with vacuum tubes containing sodium fluoride to determine plasma glucose after overnight fasting. Blood specimens were centrifuged at 4°C and 3000 rpm for 10 minutes, and the plasma was transferred and stored at −20°C for biochemical analyses. Plasma glucose was measured with a modified hexokinase enzymatic method (HTACHI automatic clinical analyzer, Model 7060, Tokyo, Japan).

### Definition of T2DM

T2DM was defined as a self-reported history of diabetes or undiagnosed diabetes after excluding type 1 diabetes mellitus, gestational diabetes mellitus, and diabetes due to other causes. According to the American Diabetes Association (ADA) diagnostic criteria [22], previously undiagnosed T2DM was defined as having fasting plasma glucose (FPG) ≥7.0 mmol/L. All participants brought their prescribed medications during the clinic visit, and a self-reported history of diabetes was confirmed by the use of insulin or oral hypoglycemic agents. In addition, the hospitalized patients with diabetes had their charts reviewed.

### Statistical analysis

Data are presented as mean ± standard deviation (sd.). For continuous and categorical variables, difference-between-group was determined by ANOVA test and chi-square (χ^*2*^) test, respectively. Univariate and multivariate logistic regression models were built to quantify the risk of undiagnosed T2DM associated with resting heart rate adjusting for potential confounding and socioeconomic variables included age, education, occupation, marital status, individual income, smoking, drinking, fat intake, vegetable and fruit intake, family history of T2DM, waist circumference, physical activity, pulse pressure, and medication use. Receiver Operating Characteristic (ROC) was used to determine the best cut-off points for sensitivity and specificity. Area under the ROC curve (AUC) was also utilized to compare the combined sensitivity and specificity among different categories of subjects. All analyses were performed using SAS 9.1 (SAS Institute, USA). All reported *P*-values were two-sided, and *P*-values of less than 0.05 were considered to be statistically significant.

## Results

Table 1 describes the general characteristics of the study sample stratified by gender. A total of 16, 636 subjects who met the criteria enrolled in the study. For male and female subjects, the mean ages (mean ± sd.) were 54.51 ± 11.06 and 52.31 ± 10.79 years, respectively, and prevalence rates of undiagnosed T2DM were 4.33% and 5.26%, respectively. The differences of the relevant parameters were compared, and the statistical significance were found about age, occupation, education level, physical activity, individual annual income, current smoking and drinking status, fat intake, vegetable and fruit intake, waist circumference, fasting glucose level, resting heart rate, and prevalence rate of undiagnosed T2DM between male and female (*P* < 0.01). However, marital status, pulse pressure, and family history of T2DM were similar between genders (*P* > 0.05).

**Table 1:** **Baseline characteristics in male and female subjects (** ***n =*** **16, 636)**

Variables	Male (*n =*6, 533)	Female (*n =*10, 103)	*P*value
Age (years), mean (±sd)	54.51(11.06)	52.31(10.79)	0.0001
Occupation, n (%)			0.0001
Farmers	4613(70.61)	9557(94.60)	
Laborers	1433(21.93)	425(4.21)	
Employers/managers	487(7.45)	121(1.20)	
Education, n (%)			0.0001
No education	420(6.43)	2461(24.36)	
Primary school	2054(31.44)	3666(36.29)	
Middle school	3101(47.47)	3371(33.37)	
High school	855(13.09)	568(5.62)	
College and above	103(1.58)	37(0.37)	
Marital status, n (%)			0.4178
Married/cohabitation	5964(91.29)	9186(90.92)	
Divorced/widowed/unmarried	569(8.71)	917(9.08)	
Physical activity, n (%)			0.0001
Low	1665(25.49)	3365(33.31)	
Moderate	1142(17.48)	2432(24.07)	
High	3726(57.03)	4306(42.62)	
Mean individual income (annual), n (%)			0.0001
< 1000 CNY	2464(37.72)	4050(40.09)	
1000 - CNY	2033(31.12)	3277(32.44)	
≥ 2000 CNY	2036(31.16)	2776(27.48)	
Current smoking, n (%)	3501(53.59)	38(0.38)	0.0001
Current drinking, n (%)	1721(26.34)	63(0.62)	0.0001
More high-fat diet, n (%)	627(9.60)	285(2.82)	0.0001
More vegetable and fruit intake, n (%)	3503(53.62)	4756(47.08)	0.0001
Family history of T2DM, n (%)	307(4.70)	510(5.05)	0.3093
Waist circumference (cm), mean (±sd)	83.17(10.10)	82.63(10.25)	0.0006
Pulse pressure (mmHg), mean (±sd)	47.89(11.62)	48.02(14.11)	0.5401
Fasting glucose (mmol/L), mean (±sd)	5.49(1.09)	5.60(1.22)	0.0001
Resting heart rate (beats/min), n (%)			0.0001
<60	706(10.81)	361(3.57)	
60 -	2115(32.37)	2344(23.20)	
70 -	2223(34.03)	4026(39.85)	
≥ 80	1489(22.79)	3372(33.38)	
Undiagnosed T2DM, n (%)	283(4.33)	531(5.26)	0.0070

*Abbreviations:* *sd* standard deviation, *CNY* ChinaYuan, *T2DM* type 2 diabetes mellitus.

Table 2 summarizes the unadjusted and adjusted odds ratios (*OR*) and 95% Confidence Interval (*CI*) of the risk of undiagnosed T2DM associated with resting pulse rate according to the four resting heart rate categories (<60, 60–69, 70–79, and ≥80 beats/min). The results showed that resting heart rate and the risk of undiagnosed T2DM had a positively dose–response effect (*P* for trend < 0.01). After adjusting for age, education, occupation, marital status, individual income, smoking, drinking, fat intake, vegetable and fruit intake, family history of T2DM, waist circumference, physical activity, pulse pressure, and medication use, the *OR* (95% *CI*) by providing the pulse rate categories staring from the reference category were 1.04 (0.57-1.87), 2.32 (1.33-4.03), and 3.66 (2.09-6.37) for male subjects, respectively. For female subjects, the *OR* (95% *CI*) were 1.05 (0.56-1.97), 1.57 (0.86-2.86) and 2.98 (1.64-5.42), respectively.

**Table 2:** **Univariate and multivariate analyses for the risk of undiagnosed T2DM associated with resting heart rate in male and female subjects (n = 16, 636)**

Variables	Male (n = 6, 533)		Female (*n =*10, 103)	
	Crude *OR*(95% *CI*)	Adjusted *OR*(95% *CI*) ^a^	Crude *OR*(95% *CI*)	Adjusted *OR*(95% *CI*) ^a^
<60	1.00	1.00	1.00	1.00
60 -	1.05 (0.58-1.88)	1.04(0.57-1.87)	0.95 (0.51-1.76)	1.05(0.56-1.97)
70 -	2.35 (1.36-4.06)	2.32(1.33-4.03)	1.37 (0.76-2.48)	1.57(0.86-2.86)
≥ 80	3.78 (2.19-6.53)	3.66(2.09-6.37)	2.47 (1.37-4.45)	2.98(1.64-5.42)
*P* for trend	<0.001	<0.001	<0.001	<0.001

^**a**^Adjusted for age, education, occupation, marital status, individual income, smoking, drinking, fat intake, vegetable and fruit intake, family history of T2DM, waist circumference, physical activity, pulse pressure, and medication use.

**Figure 1:**
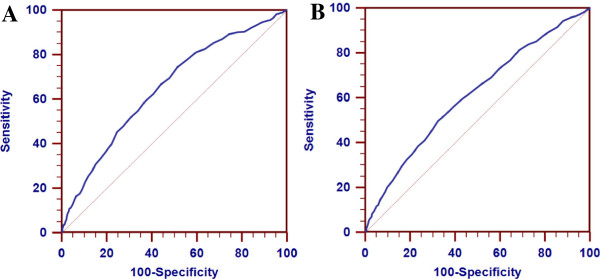
**Receiver operating characteristic (ROC) curve of resting heart rate for identifying undiagnosed T2DM in male (A) and female (B) subjects.** The areas under the ROC curves were 0.65 ± 0.02 and 0.61 ± 0.01 for male and female subjects, respectively, which suggested that resting heart rate had limited potential for screening undiagnosed T2DM.

Figure 1 presents the ROC curve of resting heart rate for identifying the risk of undiagnosed T2DM in male and female subjects. The AUC indicated that resting heart rate had a fairly poor separation for predicting those at high risk of undiagnosed T2DM in male (AUC = 0.65 ± 0.02, 95% *CI*: 0.64-0.66) and female (AUC = 0.61 ± 0.01, 95% *CI*: 0.60-0.62) subjects.

Table 3 shows the sensitivity, specificity, positive likelihood ratio (+LR), negative likelihood ratio (− LR), positive predictive value (PPV), and negative predictive value (NPV) for different levels of resting heart rate cut-off in male and female subjects. Considering the best cut-off values of resting heart rate with the highest sensitivity and specificity combinations, a resting heart rate ≥70 beats/min was found to have maximal sensitivity (74.56%) and specificity (48.64%) for predicting those at high risk of undiagnosed T2DM in male subjects. For female subjects, the optimum cut-off point for predicting those at high risk of undiagnosed T2DM was a resting heart rate ≥79 beats/min with 49.72% sensitivity and 67.53% specificity.

**Table 3:** **Resting heart rate cut-off values for predicting undiagnosed T2DM in male and female subjects (n = 16, 636)**

Resting heart rate (beats/min)	Sensitivity	Specificity	+LR	-LR	PPV	NPV
**Male**						
68	81.27	40.35	1.36	0.46	5.8	97.9
69	78.09	44.14	1.40	0.50	6.0	97.8
*70*	*74.56*	*48.64*	*1.45*	*0.52*	*6.2*	*97.7*
71	69.61	52.14	1.45	0.58	6.2	97.4
72	66.78	56.03	1.52	0.59	6.4	97.4
**Female**						
77	56.31	60.53	1.43	0.72	7.3	96.2
78	52.24	64.22	1.47	0.74	7.5	96.1
*79*	*49.72*	*67.53*	*1.53*	*0.74*	*7.8*	*96.0*
80	44.82	70.82	1.54	0.78	7.9	95.9
81	41.05	73.60	1.56	0.80	7.9	95.7

## Discussion

In this large-scale, population-based cross-sectional survey, our findings showed that fast resting heart rate was associated with a moderate risk of undiagnosed T2DM for male and female subjects. However, resting heart rate as a marker had limited potential for screening those at high risk of undiagnosed T2DM in adults living in rural areas.

Fast heart rate or tachycardia is a signal from the sympathetic nervous system, a part of the autonomic nervous system, which is the body's fight or flight response that governs instinctive responses [23–25]. Increases in sympathetic activity elevate not only pulse rate but also blood glucose, and elevated blood glucose is a specific and pathologic feature of T2DM [14]. Therefore, chronic sympathetic over-activity might underlie the development of T2DM in subjects with tachycardia [26]. Moreover, autonomic dysfunction causing tachycardia and hypertension is an established and common complication of T2DM [26–28].

A recent research from the Australian Diabetes Obesity and Lifestyle (AusDiab) Study reported resting heart rate is associated with an increased risk of diabetes over a 5-year period, particularly among non-obese men [13]. An epidemiological study from the Chicago Heart Association Detection Project in Industry Study reported that resting heart rate in middle age was positively associated with diabetes diagnosis and diabetes mortality in older age [29]. Likewise, a positive association between resting heart rate and incident T2DM was also observed in the Japanese population, and higher pulse rate might predispose to the development of obesity and T2DM [30]. However, the positive association between pulse rate and diabetes was not found in the Brazilian population [31], which could be explained by different ethical, racial, or geographic background, because resting heart rate and T2DM might be affected by their experiences of racism or concentration in particular geographical locations. In addition, a dose–response effect was also observed in the present study. Increasing resting heart rate categories were associated with a significantly increasing risk for undiagnosed T2DM (*OR* ranged from 1.05 to 3.66 in male and from 1.05 to 2.98 in female). This finding was similar to Zhang et al.’s study [16]. Overall, our findings were consistent with previous studies, and support the biological plausibility of a positive association between resting heart rate and the risk of undiagnosed T2DM.

Perhaps the most striking findings were the results of the sensitivity and specificity of resting heart rate as a marker for identifying those at high risk of undiagnosed T2DM. Indeed, sensitivity and specificity are important when testing whether a marker can accurately discriminate positive and negative outcomes [32]. The ideal indicator should have both high sensitivity and high specificity [33]. However, in our study, resting heart rate as a marker demonstrated poor sensitivity (males with 74.56%, and females with 49.72%) and specificity (males with 48.64%, and females with 67.53%) for identifying true positive or negative patients in both genders of the adults living in rural areas. With these results, resting heart rate cannot be used to accurately screen undiagnosed T2DM patients.

Since AUC provides a superior performance index in addition to superior accuracy, it is often used to evaluate the predictive accuracy of classifiers [34]. The AUC of a classifier can be defined as the probability of the classifier to rank a randomly chosen positive example higher than a randomly chosen negative example, and the higher the AUC means higher accuracy [34, 35]. This study also used AUC values for performance comparisons of different levels of resting heart rate. For male and female subjects, the AUC value indicated that resting heart rate had limited potential for screening elevated risk of undiagnosed T2DM in terms of predictive accuracy, which suggested that resting heart rate as a marker for identifying undiagnosed T2DM had fairly poor accuracy and reliability in both genders of adults living in rural areas.

Although this is the first study to explore and evaluate the feasibility of using resting heart rate as a marker for identifying the risk of undiagnosed T2DM, some limitations should be mentioned. Firstly, the cross-sectional design does not offer support to causality statements, therefore, prospective studies for different populations are necessary to describe more accurately the longitudinal relationship between resting heart rate and undiagnosed T2DM. Secondly, the absence of oral glucose tolerance tests (OGTT) to identify and confirm T2DM patient, which should be considered in future research. Thirdly, the absence of thyroid function, left ventricular function or insulin measures to evaluate the relationship between resting pulse rate and glucose metabolism should be considered in future research. Fourth, only resting heart rate was used to identify those at high risk of undiagnosed T2DM in this study, and combination with other cardiovascular risk factor could screen more adequately subjects at increased risk to develop T2DM [14]. Another possible limitation is that resting heart rate was classified into four categories. This approach was chosen because it has been applied in previous epidemiological studies [30]. Despite these limitations, the results are based on a large population-based, epidemiological study after adjusting for potential confounders, and the exposure assessment of resting heart rate has been carried out systematically in this study, which precludes differential reporting in relation to the outcome.

## Conclusions

Our findings demonstrated that fast resting heart rate was significantly associated with an increased risk of undiagnosed T2DM in both rural male and female adult population. More importantly, this data revealed that resting heart rate as a marker had limited potential for screening those at high risk of undiagnosed T2DM, which suggested that the resting heart rate might not be utilized as a perfect marker to identify the risk of undiagnosed T2DM in adults living in rural areas.

### Ethics approval

Ethics approval was obtained from the Zhengzhou University Medical Ethics Committee, and written informed consent was obtained for all participants.

### What is already known on this topic?

Fast pulse rate at rest might raise the risk for the development of type 2 diabetes mellitus (T2DM). However, data from rural areas are limited and mainly from urban areas and developed countries. In addition, no study has evaluated whether resting heart rate could be used as a marker for identifying the risk of undiagnosed T2DM.

### What does this study add?

Our findings showed that fast resting heart rate was significantly associated with an increased risk of undiagnosed T2DM in both rural male and female adult population. More importantly, these data revealed that resting heart rate as a marker had limited potential for screening those at high risk of undiagnosed T2DM, which suggested that it might not be served as a perfect marker to identify the risk of undiagnosed T2DM in adults living in rural areas.

## Abbreviations

T2DM: Type 2 diabetes mellitus
WC: Waist circumference
ROC: Receiver operating characteristic
AUC: Area under the ROC curve
OR: odds ratios
CI: Confidence interval.
